# New Insights into Crystallization of Heterophasic Isotactic Polypropylene by Fast Scanning Chip Calorimetry

**DOI:** 10.3390/polym12081683

**Published:** 2020-07-28

**Authors:** Daniela Mileva, Jingbo Wang, Markus Gahleitner, Katalee Jariyavidyanont, René Androsch

**Affiliations:** 1 Borealis Polyolefine GmbH, Innovation Headquarters, Sankt Peterstrasse 25, 4021 Linz, Austria; Jingbo.Wang@borealisgroup.com (J.W.); markus.gahleitner@borealisgroup.com (M.G.); 2 Interdisciplinary Center for Transfer-oriented Research in Natural Sciences, Martin Luther University Halle-Wittenberg, 06099 Halle/Saale, Germany; katalee.jariyavidyanont@iw.uni-halle.de

**Keywords:** polypropylene, heterophasic polypropylene, crystallization, crystal nucleation, fast scanning chip calorimetry (FSC)

## Abstract

The crystallization kinetics of metallocene-catalyzed heterophasic isotactic polypropylene composed of a matrix of isotactic polypropylene (iPP) and rubbery particles made of random ethylene–propylene copolymers (EPC), often denoted as heterophasic iPP copolymers, was analyzed as a function of the cooling rate and supercooling in nonisothermal and isothermal crystallization experiments, respectively. Fast scanning chip calorimetry (FSC) allowed assessing crystallization at processing-relevant conditions, and variation of the content (0–39 wt %) and composition (0–35 wt % propylene counits) of the EPC particles revealed qualitatively new insight about mechanisms of heterogeneous crystal nucleation. For neat iPP homopolymer, the characteristic bimodal temperature dependence of the crystallization rate due to predominance of heterogeneous and homogeneous crystal nucleation at high and low temperatures, respectively, is reconfirmed. At high temperatures, in heterophasic iPP, the here studied ethylene-(C2)-rich EPC particles accelerate crystallization of the iPP-matrix, with the acceleration or nucleation efficacy correlating with the EPC-particle content. The crystallization time reduces by more than half in presence of 39 wt % EPC particles. An additional nucleating effect of the EPC particles on iPP-matrix crystallization is detected after their crystallization, suggesting that liquid/rubbery particles are less effective than solid/semicrystalline particles in affecting crystallization of the surrounding iPP-matrix. At low temperature, homogeneous crystal nucleation in the iPP-matrix outpaces all heterogeneous nucleation effects, and the matrix-crystallization rate is independent of the sample composition. The obtained results lead to the conclusion that the crystallization kinetics of iPP can be affected significantly by the content and composition of EPC particles, even towards superfast crystallizing iPP grades.

## 1. Introduction

Since the introduction of isotactic polypropylene (iPP) in the mid-1950’s [1,2], this polymer found numerous fields of application as commodity thermoplastic and also for many engineering purposes [3,4]. Many of the favorable properties like stiffness, tensile strength, or heat resistance relate to the presence of a rather large fraction of crystalline phase; on the other hand, simultaneously, the material is characterized by relatively low ductility, toughness, or impact strength, in particular at low temperatures [5,6,7]. Reasons for such deficiency may be the phenomena of so-called cross-hatching of crystal lamellae within the semicrystalline spherulitic superstructure [8,9,10,11,12], the crystallization-induced formation of a large rigid amorphous fraction [13,14,15,16], and a rather high glass transition temperature of only slightly below room temperature, complicating strain-sensitive applications for this polymer at subambient conditions.

In order to overcome these limitations, several engineering concepts have been applied for modifying the structure and, with that, correspondingly, the property profile. This includes, among others, changing the crystallization behavior, such to replace formation of crosshatched α-crystal lamellae by only radially growing β-crystal lamellae [17,18,19,20], or enforcing the formation of smaller spherulites by addition of nucleating agents [21,22,23]. A different improvement approach of deformation-related properties by variation of the crystallization behavior is the modification of the macromolecular architecture as to deteriorate the crystallization process and reduce the crystallinity. This is achieved with the addition of 1-alkenes at random position into the propylene chain, simultaneously leading to a decrease of the glass transition temperature depending on the type and concentration of the counit [24,25,26,27,28,29].

Besides variation of the crystallization behavior of iPP, introducing a separate, particle-like rubbery phase into the iPP-matrix is a further common strategy to improve the low toughness and impact strength of neat iPP. Early developments employed externally generated ethylene–propylene or ethylene–propylene–diene rubbers, being melt blended with iPP homopolymer in a separate compounding step after synthesis [30,31,32,33,34]. More recent and sophisticated innovations included the development of so-called heterophasic copolymers from multireactor gas phase or bulk/gas phase plants, in which a semicrystalline iPP matrix is synthesized in a first step, followed by synthesis of rubbery particles in a second step, finely dispersed in the matrix. In addition, in these materials, the rubber-like phase is composed of random ethylene–propylene copolymers or pure polyethylene [34,35,36,37,38,39]. In comparison to iPP/EPC matrix–particle systems prepared by compounding, in-reactor blends offer the advantages of improved cost efficiency since a separate compounding step is not needed, as well as inherently improved compatibility between matrix and particle phase [39]. With the choice of different catalyst systems and tuning of synthesis parameters like temperature or time, a large variety of heterophasic copolymers of different structure and properties can be obtained.

Important structural parameter of heterophasic iPP copolymers are the crystalline phases of the iPP matrix as well as of the dispersed EPC. Crystallization is strongly dependent on temperature [40,41,42] and, therefore, largely affected by the conditions of processing, often involving fast cooling of the melt and solidification at high supercooling [43,44,45,46]. For neat iPP, much progress in characterization of the kinetics of crystallization was achieved by employing fast scanning chip calorimetry (FSC), allowing application of process-relevant crystallization conditions. In short, iPP shows a bimodal temperature dependence of the crystallization rate, with maxima at around 30 and 80 °C, associated to homogeneous and heterogeneous crystal nucleation, respectively [47,48,49,50]. Addition of EPC may retard the matrix crystallization process, as was found for samples containing 60 and 70 wt % EPC with a high propylene (C3) content of 49 and 70 wt %, respectively [51], or accelerate it [52]. The latter observation was obtained on a sample containing close to 30 wt % EPC with an ethylene (C2) content of around 40 wt %, however, only for crystallization at rather high temperature. FSC, allowing assessing crystallization at higher supercooling of the melt, suggested that the fast ethylene-counit crystallization process in the EPC, occurring at around 60–70 °C, slows down the iPP-matrix crystallization; at very low temperatures, it was proposed that EPR propylene-counit crystallization accelerates the iPP-matrix crystallization process again [52]. Systems containing similarly low amount of EPC around 20 wt % revealed a distinct effect of the EPR composition on the matrix spherulite growth rate, being largest at intermediate contents of ethylene and propylene, as well as revealing ethylene counit crystallization well above 100 °C if the ethylene-counit content is larger than 70 wt % [53].

Considering that crystallized EPC particles due to blocky ethylene or propylene sequences exhibit different properties in comparison to fully amorphous particles or an iPP matrix with a semicrystalline morphology altered by EPC nucleation effects, both affecting ultimate properties, analysis of the correlation between the synthesis-controlled molecular architecture and the rather complex crystallization of such heterophasic copolymers is well justified. The above examples of such crystallization studies [51,52,53] employed samples prepared using classical Ziegler–Natta catalyst systems, leading to well-known effects on the intra- and intermolecular homogeneity of the counit incorporation into the random copolymer as well as on the molar-mass distribution [54,55]. However, recently, also the use of metallocene-based single-site catalyst systems was discussed with dedicated investigation of the crystallization behavior completely absent. For this reason, in the present work, an attempt is made to analyze crystallization of both iPP-matrix and ethylene-rich EPC particles prepared by metallocene catalysts. In advance to the recent FSC study of a specific Ziegler–Natta catalyzed heterophasic copolymer, here a set of samples of different EPC content on one side and different, but always rather high, ethylene content on the other side is available, allowing systematic detection of their effects on the crystallization behavior.

## 2. Materials and Methods

### 2.1. Materials

All polymers of the present study were synthesized using an emulsion-type single-site metallocene catalyst, described in detail elsewhere [56], employing a batch reactor operated in two-stage mode. In a first step, the polypropylene matrix was formed in a liquid bulk phase at a temperature of 80 °C, and then, the particle-like ethylene–propylene copolymer phase was polymerized in gas phase at slightly lower temperature of 70 °C. By variation of the feed of ethylene and propylene, as well as reaction times and temperatures of the bulk and gas phase reaction steps, it was possible generating samples of different content of EPC particles, which in addition contained different amounts of ethylene. Table 1 provides relevant information about the molecular characteristics of the samples used. Sample names (PP/EPC-x-y) contain information about the percentage EPC content (x) and the ethylene (C2) content in the EPC (y), both given in wt %. As such, the study includes a reference sample PP/EPC-0-0, that is, a homopolymer not containing EPC, and three heterophasic copolymers with approximately similar total amount of around 25 wt % ethylene. Variables in these copolymers are the EPC content of 27, 29, and 39 wt %, with ethylene contents in the EPC particles of 90, 79, and 65 wt %, respectively. Such high ethylene-unit content classifies the EPC as close to a linear-low-density polyethylene with few –CH_3_ side groups. The polymer powders were stabilized with 1500 ppm of Irganox B 225FF as antioxidant and 500 ppm of calcium stearate as acid scavenger in a twin-screw Prism TSE 16TC extruder (Thermo Electron Corp., Staffordshire, UK), applying a temperature profile from hopper to die of 170-190-210-220-200 °C, throughput of 2.5 kg/h, and a screw speed of 180 rpm.

### 2.2. Instrumentation

For analysis of the crystallization behavior of the samples listed in Table 1, we employed a power-compensation differential scanning chip calorimeter Flash DSC 1, provided by Mettler Toledo (Greifensee, Switzerland). The main instrument was connected to a Huber TC100 Intracooler (Offenburg, Germany), allowing setting the sensor-support temperature to −90 °C for experiments involving rapid cooling. The sample environment was purged with nitrogen gas at a flow rate of 40 mL/min. Samples were prepared by microtoming sections with a thickness of 8 µm from the available pellets, employing a SLEE rotary microtome (Mainz, Germany). The lateral size of the sections was then reduced to 50–100 µm using a scalpel and a stereomicroscope, before placing them onto the membrane of the UFS 1 sensor. With the knowledge of the lateral temperature-distribution of the particular sensor, attention was paid to use only its central area where the temperature profile is homogeneous [57]. For improvement of the thermal contact between the sensor membrane and the sample, either silicon oil or a small piece of gold leaf on a thin silicone-oil film was employed as contact medium, effectively allowing for shrinkage and expansion of the samples without straining the membrane [58]. Before placing the samples to the sensor, the latter was conditioned and temperature corrected according to the instrument operating instructions. FSC was used to analyze crystallization on cooling between 1 and 3000 K/s and at temperatures between 0 and 110 °C.

Complementary differential scanning calorimetry (DSC) experiments were performed using a Mettler Toledo heat-flux DSC 1 attached to a Huber TC100 Intracooler. The mass of the samples was about 4.5 mg, and for encapsulation, 20 µL aluminum pans were employed. The furnace was purged with nitrogen, using a flow rate of 60 mL/min.

## 3. Results and Discussion

### 3.1. Nonisothermal Crystallization

**Figure 1:**
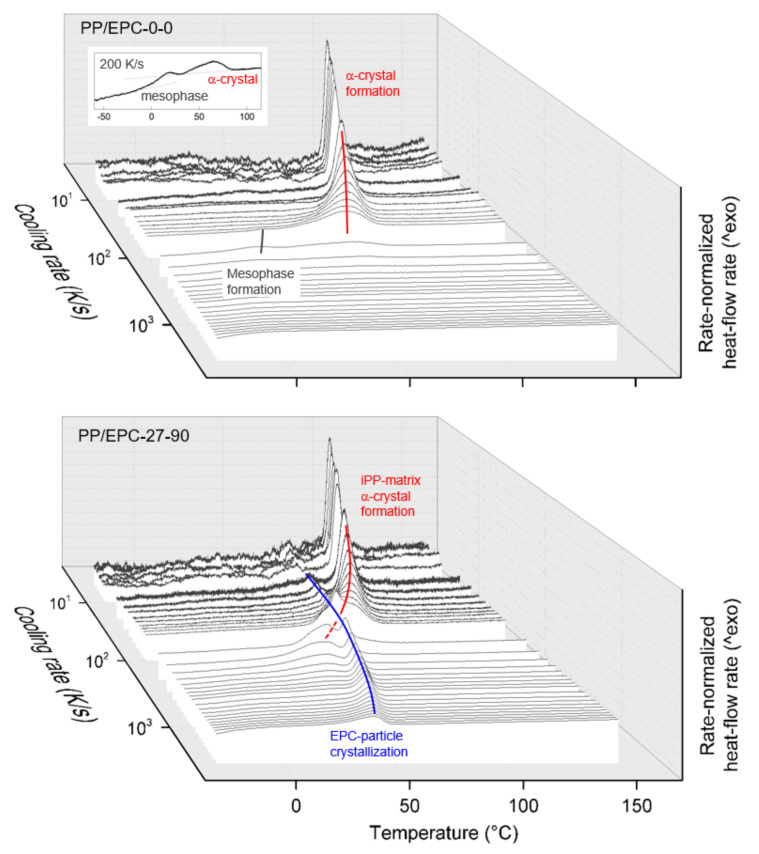
Sets of rate-normalized FSC cooling curves of the samples PP/EPC-0-0 (iPP homopolymer) (top) and PP/EPC-27-90 (heterophasic iPP containing 27 wt % EPC particles with 90 wt % ethylene counits) (bottom). Exothermic heat-flow rate is directed upwards.

Figure 1 shows sets of rate-normalized FSC cooling curves of the samples PP/EPC-0-0 (iPP homopolymer, top) and PP/EPC-27-90 (bottom), serving as examples of the performed nonisothermal crystallization experiments. Exothermic heat-flow is directed upwards, and the various phase transitions are indicated/labelled using red-, gray-, and blue-color coding for formation of α-crystals of iPP, mesophase formation of iPP, and crystallization of the EPC particles, respectively. It is worth emphasizing that the signal-to-noise ratio in FSC scanning experiments decreases with decreasing rate of temperature change, similar as in conventional DSC [59,60,61], which explains the lowered data quality in experiments involving cooling slower than 10 K/s.

Regarding the sample PP/EPC-0-0, that is neat iPP, slow cooling at rates up to around 200 K/s allows the formation of α-crystals, with the crystallization temperature decreasing with cooling rate. If high-temperature α-crystal formation is incomplete, then further cooling permits formation of mesophase at around room temperature, though the transition enthalpy of the mesophase formation process is rather low. The inset at the back plane of the graph shows the 200-K/s-cooling curve enlarged for improved presentation of the mesophase-formation process, which at the low-temperature side, smoothly overlaps the glass transition. When cooling the melt faster than few hundreds kiloseconds to below the glass transition temperature, any ordering process is suppressed. The experimental observations obtained on neat iPP further confirm FSC studies available in the literature [50,62,63,64], however, they are still shown here for comparison with the nonisothermal crystallization behavior of heterophasic iPP, synthesized using similar conditions.

Regarding the sample PP/EPC-27-90, which is the heterophasic iPP consisting of an iPP-matrix containing 27 wt % EPC particles of high C2 content of 90 wt %, separate matrix- and particle-crystallization events are detected. The iPP-matrix crystallizes qualitatively similar as in neat iPP, at least in case of low cooling rates up to around 100 K/s (see solid red line). Upon faster cooling, however, mesophase formation at around room temperature is not observed but minor high-temperature crystallization associated with formation of α-crystals (see dashed red line). The critical cooling rate to suppress ordering processes in the matrix, still, is few hundreds K/s. EPC-particle crystallization in a LLDPE-like mode occurs on slow cooling at around 75 °C, with the peak temperature showing only a weak dependence on the cooling rate (see blue line labeled “LLDPE particle crystallization”). Furthermore, in contrast to the crystallization peak attributed to crystallization of the iPP-matrix, the area of the EPC-crystallization peak seems independent on the cooling rate within the analyzed cooling rate range up to 3000 K/s.

**Figure 2:**
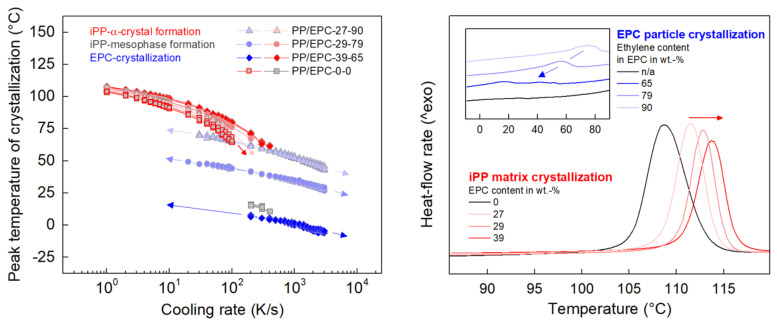
Peak crystallization temperature of iPP homopolymer and heterophasic iPP as a function of the cooling rate (**left**). DSC cooling scans, heat-flow rate as a function of temperature, of iPP and heterophasic iPP (**right**). Red, gray, and blue coloring of data refer to crystallization of iPP, mesophase formation of iPP, and crystallization of EPC particles, respectively. In the right graph, exothermic heat-flow rate is directed upwards, and the cooling rate was 20 K/min (0.33 K/s).

Quantitative information about the cooling-rate dependence of FSC transition temperatures of all samples shows the left graph in Figure 2. The right plot additionally shows DSC scans, confirming observations obtained by FSC as well as providing information about crystallization on very slow cooling of 20 K/min (0.33 K/s). In analogy to color coding of phase transitions in Figure 1, red, gray, and blue coloring of data indicate formation of iPP-α-crystals, of the mesophase of iPP, and of crystals in the EPC particles, respectively.

In addition to information gained by visual inspection of the cooling curves in Figure 1, Figure 2 leads to two further important conclusions. First, the crystallization temperature of the iPP-matrix in heterophasic samples is slightly higher than in case of neat iPP. Although at low cooling rates the difference is rather small (being less than 10 K for all samples), the difference increases with cooling rate and exceeds 10 K in particular when the EPC content is rather high. Moreover, a systematic effect of the EPC content on iPP-matrix crystallization is detected, such that the crystallization temperature increases with the EPC content. It appears that the EPC particles act like heterogeneous nucleation sites promoting crystallization of the iPP-matrix. Second, the data of Figure 2, both FSC (left) and DSC (right), suggest a correlation between the ethylene counit content in the EPC particles and the crystallization temperature (see blue data). The higher the ethylene counit content, in other words, the lower the number of CH_3_-groups/branches in the LLDPE-like macromolecules, the higher is the crystallization temperature. This finding is in accord with studies of the crystallization behavior of ethylene-rich random ethylene–propylene copolymers having revealed, e.g., inclusion of methyl groups into polyethylene crystals with orthorhombic or pseudohexagonal symmetry depending on methyl group concentration in the chains, as well as decreasing melting temperature and crystallinity with increasing methyl group content [65,66,67,68].

**Figure 3:**
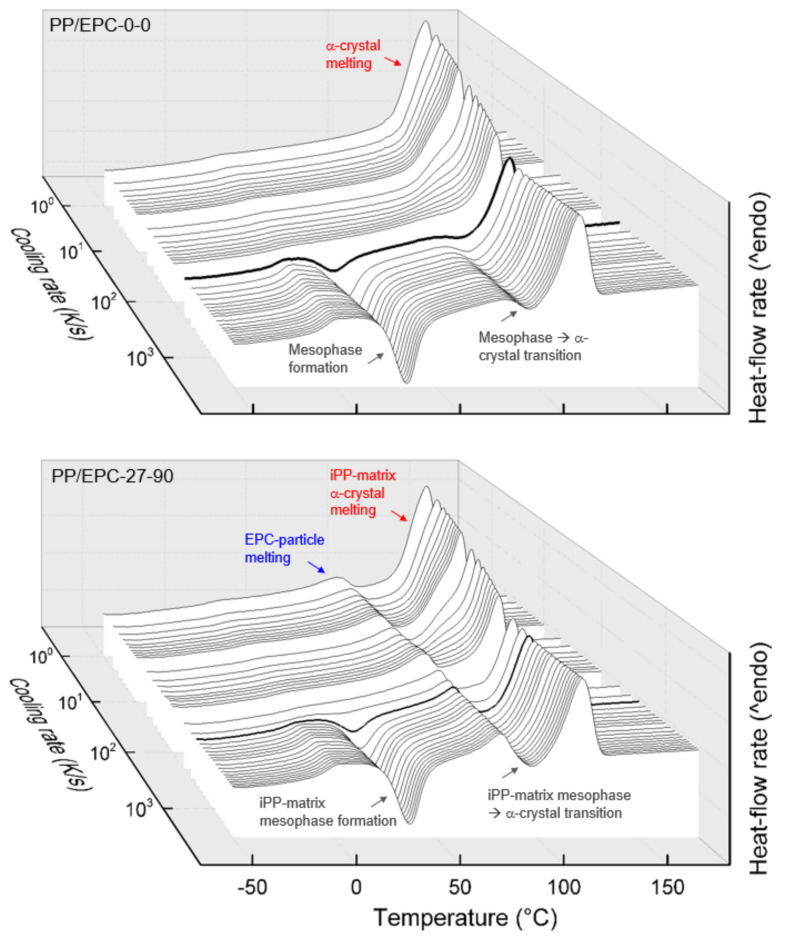
Sets of FSC heating curves of the samples PP/EPC-0-0 (iPP homopolymer, top) and PP/EPC-27-90 (heterophasic iPP, bottom). Exothermic heat-flow rate is directed downwards and the heating rate is 1000 K/s. Color coding of the various phase transitions is in analogy to Figure 1 and Figure 2. Bold curves in the top and bottom plots refer to prior cooling at rates of 200 and 400 K/s, respectively.

Figure 3 shows sets of FSC heating curves of the samples PP/EPC-0-0 (iPP homopolymer, top) and PP/EPC-27-90 (heterophasic iPP, bottom), recorded using a heating rate of 1000 K/s, after prior cooling the melt at different rates between 3000 K/s (front curve) and 1 K/s (back curve). Exothermic heat-flow is directed downwards, and the various phase transitions are indicated/labelled using red, gray, and blue colors, in analogy to Figure 1 and Figure 2. Again, the data of Figure 3 serve as examples demonstrating the different crystallization and melting behaviors of neat iPP and heterophasic iPP. In case of the homopolymer (PP/EPC-0-0), fast cooling completely suppressed crystallization and ordering processes (see front curves in Figure 1). Subsequent heating to above the glass transition temperature first causes exothermic mesophase formation around room temperature, followed by exothermic transformation of the mesophase into α-crystals slightly below 80 °C, and finally endothermic melting of the crystals formed during heating. Details of the various transitions are reported in the literature [69,70,71,72,73,74,75,76] and not repeated here since being out of the scope. Decreasing the cooling rate to below about few hundreds K/s allows crystallization and ordering during cooling, and consequently, on subsequent heating, cold-crystallization/mesophase formation at around 25 °C is reduced (see bold-drawn heating curve after prior cooling at 200 K/s). Even slower cooling increasingly permits completion of crystallization during cooling, and on heating, only melting of α-crystals formed during cooling is detected.

Qualitatively similar behavior is detected for the iPP-matrix in the heterophasic sample PP/EPC-27-90. As concluded already from the data of Figure 2, also the heating scans reveal slightly faster crystallization on cooling in case of the heterophasic sample. As demonstrated with the bold-drawn heating curves, cold-crystallization (more precise cold-mesophase-formation), being indicative of the amount of crystals formed on prior cooling, fades in the heating scans if prior cooling was performed at 400 K/s in heterophasic iPP and only around 200 K/s in neat iPP. In addition to the iPP-matrix related transitions, additional melting occurs around 70–80 °C, associated to LLDPE-crystals formed in the EPC-particle phase. This particular melting event seems nearly independent on the rate of prior cooling, regarding both its temperature position and area.

**Figure 4:**
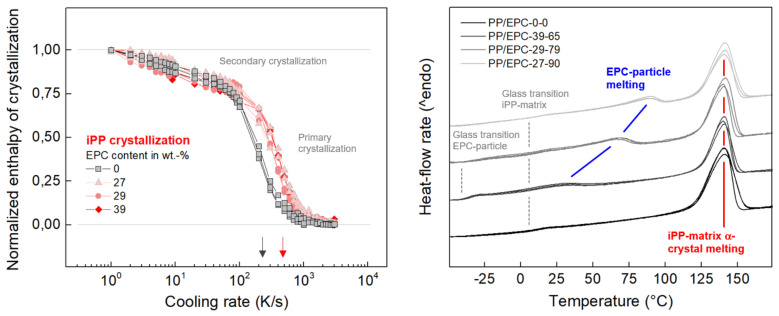
Normalized enthalpy of crystallization of neat iPP (gray squares) and of the iPP-matrix in heterophasic iPP (red symbols) as a function of the cooling rate (**left**). FSC heating scans of neat and heterophasic iPP (**right**). The heating rate is 1000 K/s, and the rate of prior cooling is 1 K/s.

Figure 4 shows with the left graph the cooling-rate dependence of the enthalpy of crystallization for neat iPP (gray squares) and the iPP-matrix in heterophasic samples (red symbols). The data were obtained by integrating the FSC-heating curves covering the iPP cold-crystallization event, the mesophase-to-crystal transition, as well as final melting. Integration also included the LLDPE-melting peak due to difficulties separating its contribution, however, since it is a cooling-rate independent constant offset only, it does not affect the discussion of the cooling-rate-dependent crystallization of iPP. Furthermore, in order to accommodate absent knowledge of the mass of FSC samples, data were (0,1)-normalized. The data further quantify improved crystallization of iPP in presence of the EPR particles, as the critical cooling rate above which crystallization/ordering is fully suppressed increases from roughly 200 K/s in neat iPP to 400 K/s in heterophasic iPP, when using the inflection point related to primary crystallization for benchmarking (see arrows at the cooling-rate axis). Though a distinction between the various heterophasic samples cannot be done, the data confirm the accelerating effect of EPC particles on iPP crystallization as the observation is in accord with the increase of the crystallization temperature in cooling experiments (see Figure 2 left).

The right plot in Figure 4 shows FSC heating scans of neat and heterophasic iPP, recorded at a rate of temperature change of 1000 K/min. Prior to heating, the samples were crystallized on slow cooling at a rate of 1 K/s. These data mainly serve for comparison of the temperatures of melting of the EPC-particle phase (LLDPE), being dependent on the molecular architecture of the ethylene–propylene copolymers. The FSC scans reveal that the melting temperature of crystals formed in EPC particles scales with the ethylene counit content such that higher concentration leads to a higher transition temperature, as indicated with the blue lines. This result is expected from former analysis of crystallization temperatures (see Figure 2, left), having revealed a similar effect of the ethylene counit content. In simple words, propylene counits deteriorate formation of crystals composed of ethylene units, though the calorimetric data do not provide information whether the propylene counits are included in the crystals as defects or not. In any case, the larger the propylene content in the copolymers, the shorter the maximum ethylene-sequence length and the smaller and more defective are the crystals, with both effects, size and perfection, affecting the melting temperature [77,78,79]. The melting peak of propylene-unit based crystals in the iPP-matrix, in contrast, is unaffected by the EPC particles (see red line). Furthermore, though out of focus, the heating scans provide rough information about glass transition temperatures in the investigated systems. All samples reveal a glass transition slightly above 0 °C, attributed to the amorphous iPP phase in both neat iPP and the matrix in heterophasic iPP. For heterophasic iPP containing 39 wt % EPC particles (PP/EPC-39-65), a further glass transition occurs at around −30 °C, presumably involving noncrystallized random ethylene–propylene copolymer in the EPR particles.

### 3.2. Isothermal Crystallization

**Figure 5:**
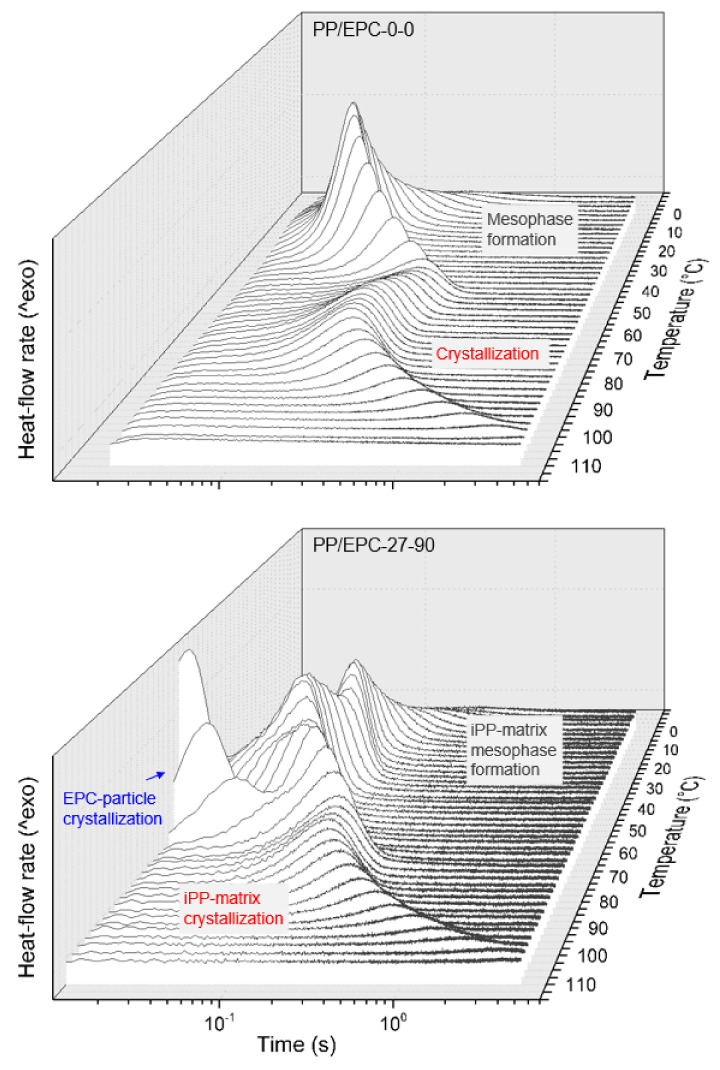
Sets of FSC crystallization isotherms of the samples PP/EPC-0-0 (neat iPP, top) and PP/EPC-27-90 (heterophasic iPP, bottom). Exothermic heat-flow rate is directed upwards and color coding of labels is in analogy to above figures.

Isothermal crystallization experiments were performed to obtain quantitative information about characteristic times of formation of the various crystalline/ordered phases. Figure 5 shows sets of FSC crystallization isotherms of the samples PP/EPC-0-0 (neat iPP, top) and PP/EPC-27-90 (heterophasic iPP, bottom), again, serving as examples, as measurements on all samples of Table 1 were performed three times. The analysis of neat iPP reveals, as expected, high-temperature α-crystal formation and low-temperature mesophase formation associated to heterogeneous and homogeneous nucleation mechanisms, respectively [42,47,71,80,81]. Crystallization is fastest at around 75 °C, whereas maximum rate of mesophase formation is observed at around ambient temperature. More complex crystallization patterns are detected for heterophasic iPP. Although high-temperature crystallization and low-temperature mesophase formation occur as in neat iPP, at intermediate crystallization temperatures between 50 and 70 °C in case of the particular sample PP/EPC-27-90, multiple crystallization events occur at identical temperature. Detecting several crystallization events in sequence at constant temperature, in general, may be caused by polymorphic transitions, as recently discussed for poly(butylene naphthalate) [82,83,84], or by independent crystallization at different rates in different phases. For heterophasic iPP, the latter reason is favored due to the presence of different crystallizable phases—iPP-matrix and EPC particles—And detection of a systematic decrease of the temperature range in which multiple events occur at identical temperature, related to the ethylene content in the EPC particles and nonisothermal crystallization temperature. Next, the crystallization isotherms, exemplarily shown in Figure 5, are evaluated regarding crystallization peak times, providing an advantageous representation of the temperature dependence of the crystallization kinetics.

**Figure 6:**
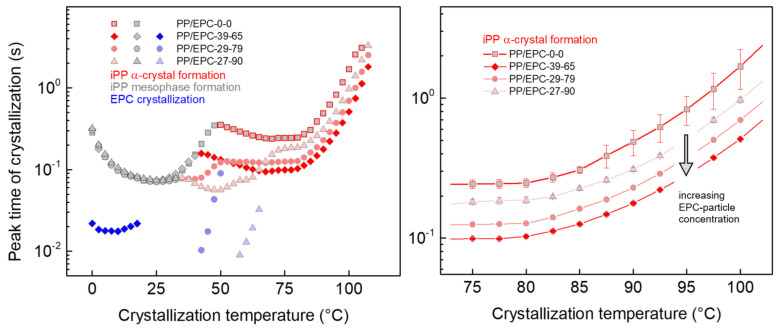
Peak time of crystallization of neat and heterophasic iPP as a function of the crystallization temperature in the entire analyzed temperature range between 0 and 110 °C (**left**) and in the high-temperature region above 75 °C (**right**). Color coding of symbols/lines is in analogy to above figures.

Figure 6 shows in the left plot peak times of crystallization for neat iPP and heterophasic iPP as a function of temperature. For easy assignment of the various phase transitions, as before, red, gray and blue colors denote the transition of the melt of iPP into α-crystals, of the melt of iPP into mesophase, and of crystallization of ethylene sequences in the EPC particles, respectively. For neat iPP, the typical bimodal temperature dependence of the crystallization rate is reproduced [42,47,71,80,81]. At high temperature (red/gray squares), heterogeneous crystal nucleation connected with formation of lamellar α-crystals dominates, whereas at low temperature (gray squares) homogeneous crystal nucleation, connected with formation of a nodular mesophase, prevails [85,86,87,88], with the predominance of the respective nucleation schemes changing at around 50 °C.

The rather complex crystallization behavior in case of heterophasic iPP may be categorized by recognizing three different temperature ranges in which there are observed systematic effects of the presence of the EPC particles. Regarding crystallization of the iPP-matrix, for the samples PP/EPC-29-79 and PP/EPC-27-90, a trimodal distribution of the crystallization rate is seen, with an additional crystallization-rate maximum observed in between those related to α-crystal formation and mesophase formation in neat iPP; only few similar observations of trimodal temperature dependencies of crystallization rates are reported in the literature [52,89,90,91]. At high temperature, e.g., above 75 °C, crystallization within the analyzed time period of 5 s only occurs in the iPP-matrix; crystallization in the EPC particles proceeds at much lower temperature only (see blue symbols in Figure 2 and Figure 6). The particular temperature range above 75 °C is replotted enlarged to the right in Figure 6, revealing a distinct and systematic effect of the EPR-particle concentration on the iPP-matrix-crystallization rate. As emphasized with the downward directed arrow, the crystallization peak time decreases with increasing EPR-particle content such that presence of almost 40 wt % EPR more than halves it. This observation consistently confirms nonisothermal crystallization experiments, which revealed the nucleating effect of the EPC particles by the increase of the crystallization temperature (see red symbols in Figure 2). Worth noting, data shown in both the left and right plots of Figure 6 represent averages of three independent measurements, with error bars inserted in the right graph, only in case of neat iPP being larger than the symbol size.

At temperatures lower than about 70 and 50 °C, in case of the samples PP/EPC-27-90 and PP/EPC-29-79, respectively, the crystallization rate increases compared to the high-temperature crystallization process and passes an addition maximum before the homogeneous nucleation process (see gray symbols) becomes effective. The increase of the crystallization rate at 70 and 50 °C in these samples correlates with crystallization in the EPC particles, represented with blue symbols/lines. It appears that if crystallization in the EPC particles occurs before crystallization in the iPP-matrix (see intersection of the corresponding red and blue data sets), then the latter process accelerates. Obviously, the nucleation efficiency of the EPC particles on crystallization of the iPP-matrix is twofold, depending whether being in liquid or solid state. Although the nucleation efficiency of noncrystallized EPC particles on iPP-matrix crystallization is illustrated with the right plot of Figure 6, crystallization of the EPC particles causes an additional increase of the crystallization rate. A possible reason might be the EPC-crystallization-induced decrease of the particle volume and generation/transfer of stress into the surrounding matrix, supported by the rather strong interfacial particle-matrix adhesion in such heterophasic iPP. For the sample PP/EPC-39-65, the above-described nucleation effect of semicrystalline EPR particles is undetectable since the heterogeneous nucleation process in the iPP-matrix is paced out by much faster homogeneous nucleation (see intersection of the blue line with gray symbols on one side and with the red dashed line on the other side).

Low-temperature crystallization of both neat iPP and the iPP-matrix in heterophasic samples proceeds via homogeneous nucleation, as well as with decreasing crystallization temperature, α-crystal growth in the respective samples is slower than the characteristic time of formation of a large number of homogeneous crystal nuclei. Note that the density of homogeneous nuclei typically, including iPP, is orders of magnitude higher than the density of heterogeneous nuclei, allowing for fast completion of crystallization [92] (see gray symbols). Worth noting that the changeover temperature between predominance of heterogeneous and homogeneous nucleation decreases to lower temperature with increasing ethylene-sequence length/crystallization temperature of the random copolymers of the EPC particles, from around 50 °C in neat iPP to 30 °C if the ethylene counit content is 90 wt %.

## 4. Conclusions

Nonisothermal and isothermal DSC and FSC experiment provide a consistent picture about the effects of the EPC-particle-concentration and -composition on the crystallization behavior of heterophasic iPP. Within the investigated range of EPC-particle concentrations from 0 to 39 wt %, the rate of high-temperature crystallization of the iPP matrix scales with the particle content, even if the particles are noncrystalline/rubbery. Crystallization of the ethylene-rich random ethylene–propylene copolymers in the particles causes an additional nucleating effect on the crystallization of the iPP matrix, suggesting a strong influence of the matrix–particle interfacial energy on the matrix crystallization behavior. As the crystallization temperature of the EPC particles is controlled by the ethylene concentration in the ethylene–propylene copolymers, variation of the chemical architecture of the EPC-molecules offers an advantageous tool for controlling the matrix crystallization behavior, in particular, at intermediate supercooling of the melt. At high melt-supercooling, homogeneous crystal nucleation in the iPP-matrix outpaces all heterogeneous nucleation effects, and the matrix-crystallization rate is independent on the presence of EPC particles. The results of the performed study lead to the conclusion that the crystallization kinetics of iPP can significantly be tailored by both the content and the composition of EPC particles.

## Figures and Tables

**Table 1:** Molecular Characteristics of the Samples of the Present Study.

Polymer	C2 (Total) ^1^ [wt %]	XCS ^2^ [wt %]	C2 (XCS) ^3^ [wt %]	IV (XCS) ^4^ [dL/g]	Matrix MFR ^5^ [g/(10 min)]
PP/EPC-0-0	0	–	–	–	35
PP/EPC-27-90	23	27	90	0.91	~35
PP/EPC-29-79	27	29	79	3.46	~35
PP/EPC-39-65	25	39	65	2.26	80

^1^ total ethylene content; ^2^ XCS = xylene soluble fraction, representing EPC content; ^3^ ethylene content in XCS (EPC), measured by Fourier-transform infrared spectroscopy; ^4^ IV = intrinsic viscosity of XCS, representing the EPC molar mass, determined in decalin at 135 °C; ^5^ MFR = melt-flow rate (230 °C, 2.16 kg) of matrix, representing the molar mass of the matrix.
